# Improvement of the Dynamic Responses of Heart Rate Variability Patterns after Needle and Laser Acupuncture Treatment in Patients with Burnout Syndrome: A Transcontinental Comparative Study

**DOI:** 10.1155/2013/128721

**Published:** 2013-10-09

**Authors:** Gerhard Litscher, Cun-Zhi Liu, Lu Wang, Lin-Peng Wang, Qian-Qian Li, Guang-Xia Shi, Ingrid Gaischek, Daniela Litscher, Xiao-Min Wang

**Affiliations:** ^1^Stronach Research Unit for Complementary and Integrative Laser Medicine, Research Unit of Biomedical Engineering in Anesthesia and Intensive Care Medicine and TCM Research Center Graz, Medical University of Graz, Auenbruggerplatz 29, 8036 Graz, Austria; ^2^Acupuncture and Moxibustion Center, Beijing Hospital of Traditional Chinese Medicine Affiliated to Capital Medical University, No. 23 Meishuguanhou Street, Beijing 100010, China; ^3^Department of Neurobiology, Capital Medical University, Beijing 100069, China

## Abstract

We investigated manual needle and laser needle acupuncture as a complementary therapy for patients with burnout syndrome. Twenty patients with a mean age ± SD of 38.7 ± 8.4 years were assigned to two groups, each consisting of ten patients. One group was treated with manual needle acupuncture and the other with laser needle acupuncture. Heart rate, heart rate variability (HRV), and a new score called dynamic acupuncture treatment score (DATS) served as evaluation parameters. The study documented significant effects on heart rate after needle acupuncture treatment and significant effects on HRV caused by both needle and laser needle acupuncture. Based on new neurovegetative acupuncture treatment evaluation scores, it can be stated that both noninvasive laser needle acupuncture and manual needle acupuncture have the potential to be a powerful approach for evidence-based complementary treatment of patients with burnout syndrome. Further transcontinental studies to verify or refute the preliminary findings are in progress.

## 1. Introduction

In the year 1974, Freudenberger defined the “Burnout syndrome” as a state of physical and mental exhaustion which develops slowly from continuous stress and use of energy to exhaustion because of excessive demands [1]. Since Freudenberger's “Staff Burnout,” published in 1974, burnout has become a synonym for psychosomatic and psychological symptoms and social consequences of a long-lasting workload exceeding an individual's capacity [1–3].

Due to unclear diagnosis, the plurality of symptoms, and diverse reasons for burnout, there are uncertainties in the literature regarding its therapy [2].

Fatigue is a common complaint mainly in the working population, with a reported prevalence varying from 7 to 45% [4–6]. It can be understood as a continuum, ranging from mild to severe, disabling fatigue, as in the chronic fatigue syndrome [4]. The phenomenon of burnout is conceptually linked with fatigue [7]. However, it is important to realize that persistently fatigued workers are not burnt out by definition and that burnt out workers might not experience fatigue as one of their major complaints [7].

Both persistent fatigue and burnout are reported to be serious conditions, but little research is available on their clinical features. In this respect, chronic fatigue syndrome has received more attention. It is characterized by persistent, medically unexplained fatigue for at least six months [7].

At the moment, not only psychotherapy, especially cognitive behavioral therapy, phytotherapy, and physiotherapy, but also complementary treatments such as acupuncture are used to treat fatigue, burnout, and chronic fatigue syndrome (see Table 1).

For the present study, patients with burnout syndrome were treated with manual needle acupuncture or laser needle acupuncture at the Beijing Hospital of Traditional Chinese Medicine (TCM) affiliated to Capital Medical University. The dynamic responses of heart rate (HR) and its variability (HRV) patterns were analyzed with respect to the different modalities of the acupuncture therapy applied. Analysis was performed at the TCM Research Center at the Medical University of Graz. This is a further clinical transcontinental teleacupuncture study between the two centers in Europe and China [8].

## 2. Patients and Methods

### 2.1. Patients

Twenty patients (10 females, 10 males; mean ages ± SD 38.7 ± 8.4 years; ranges 25–57 years) suffering from burnout syndrome and therefore receiving manual needle (group A) or laser needle (group B) acupuncture treatment were investigated at the Beijing Hospital of TCM affiliated to Capital Medical University. The clinical evaluation of the patients was performed immediately before the first HRV data recording by Chinese experts. The patients were randomly assigned to group A or B. In group A (*n* = 10; 6 females, 4 males), mean age ± SD was 39.2 ± 7.8 years (range: 29–51 years; median: 40 years); in group B (*n* = 10; 4 females, 6 males) it was 38.2 ± 9.0 years (range: 25–57 years; median: 39 years). No patient was under the influence of centrally active medication or had a history of heart or cerebrovascular disease, respiratory or neurological problems, or hypertension. The study was approved by the Ethic Committee of the Beijing Hospital of TCM and carried out in compliance with the Declaration of Helsinki. All patients gave oral informed consent. 

### 2.2. Teleacupuncture between China and Europe

Electrocardiographic (ECG) registration is performed using three adhesive electrodes (Skintact Premier F-55; Leonhard Lang GmbH, Innsbruck, Austria) which are applied to the chest. The duration of RR-intervals is measured during a special time period (5 min), and on spectral analysis basis HRV is determined.

The researchers at the Beijing Hospital of TCM used a medilog AR12 HRV (Huntleigh Healthcare, Cardiff, UK) system from the TCM Research Center at the Medical University in Graz. The system has a sampling rate of 4096 Hz [9, 10]; the raw data are stored on a memory card, and, after removing the card from the portable system, the data were read by a card reader connected with a standard computer in China and then transferred to the center in Graz via internet. The ECG data were analyzed and HRV was displayed in a way to help to judge the function of the autonomic nervous system. Similar to previous teleacupuncture studies [11–15], mean HR, total HRV, and the LF (low frequency)/HF (high frequency) ratio of HRV were chosen as preliminary evaluation parameters, as such being recommended by the Task Force of the European Society of Cardiology and the North American Society of Pacing and Electrophysiology [16]. In addition to these standard parameters, the so-called HR-HRV “dynamic acupuncture treatment score” (DATS) is introduced in this paper. DATS is defined as the ratio of HR or HRV changes at the end (post) of the acupuncture treatment in comparison to the HR or HRV alterations at the beginning (pre) of the acupuncture treatment (DATS = HRV changes post/HRV changes pre). The new score should be an estimate value to describe the dynamic effects of acupuncture treatment on HR and HRV.

### 2.3. Needle and Laser Acupuncture and Procedure

For manual acupuncture stimulation, sterile single-use needles (length: 30 mm, diameter: 0.3 mm; Huan Qiu, Suzhou, China) were inserted perpendicularly in the skin to standard depths at the acupoints. The needles were stimulated clockwise and counterclockwise for 15 seconds each, with two rotations per second, resulting in 30 rotations per stimulation. The stimulation was performed immediately after inserting the needle, 10 minutes later, and before removing the needle.

**Figure 1:**
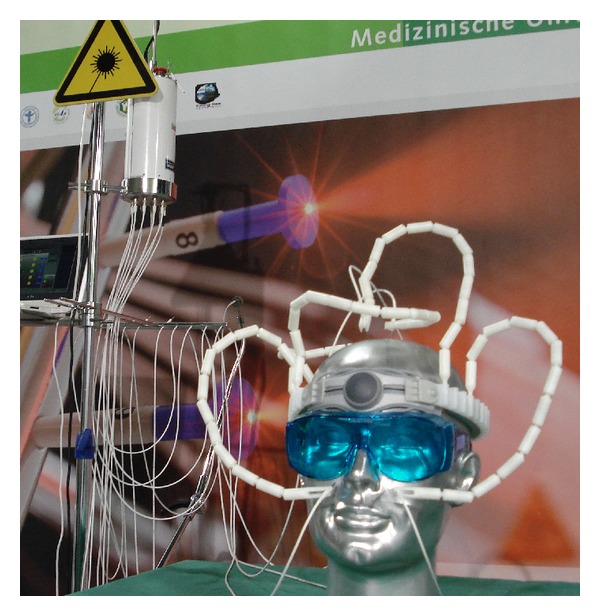
Multichannel laser acupuncture using bichromatic laser needles with red and infrared light.

Laser acupuncture was performed using laser needles [17, 18]. The first bichromatic laser needles (685 nm and 785 nm) were developed at the University of Paderborn, Germany (Dr. Detlef Schikora), and the first clinical investigations were performed in Lauenförde, Germany (Dr. Michael Weber). The first scientific experiments and publications in this field of research started in 2002 at the Medical University of Graz, Austria [17, 18]. A laser needle acupuncture system based on red and infrared light is shown in Figure 1.

Multichannel laser needle acupuncture allows the noninvasive simultaneous stimulation of individual point combinations. The system consists of flexible optical light fibers which conduct the laser light with minimal loss to the laser needle. Thus, a high optical density can be achieved at the distal end of the laser needle. The intensity of the laser needles is optimized in such a way that the patient does not immediately feel the activation of the needle (30–40 mW per needle; diameter: 500 *μ*m; duration: 20 min). More details regarding this method are described in previous studies and books [17–19].

Acupuncture points were selected by experienced Chinese acupuncturists. Each patient underwent altogether six acupuncture sessions. The interval between the treatment sessions was 2 to 4 days.

**Figure 2:**
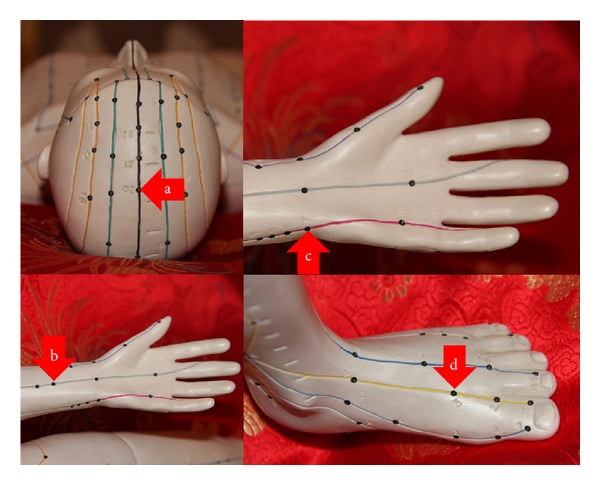
Acupuncture points used in the present study in burnout patients.

The following acupoints were used in this study (needle and laser needle acupuncture): Baihui (GV20), Neiguan (PC6), Shenmen (HT7), and Taichong (Liv3) (see also Figures 2(a)–2(d)).

**Figure 3:**
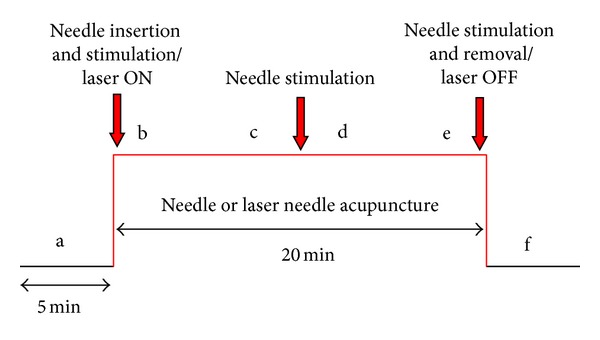
Experimental protocol for manual needle acupuncture and laser acupuncture.

The measurement profile and measurement phases (a–f; 5 min each) are shown schematically in Figure 3. Six measurement periods were compared: one before stimulation (a), four during acupuncture (b–e), and one after acupuncture (f).

### 2.4. Statistical Analysis

Data were analyzed using SigmaPlot 11.0 software (Systat Software Inc., Chicago, USA). Graphical presentation of results uses box plot illustrations. Testing was performed with repeated measures ANOVA on ranks and Tukey or Holm-Sidak test. The criterion for significance was *P* < 0.05.

## 3. Results

**Figure 4:**
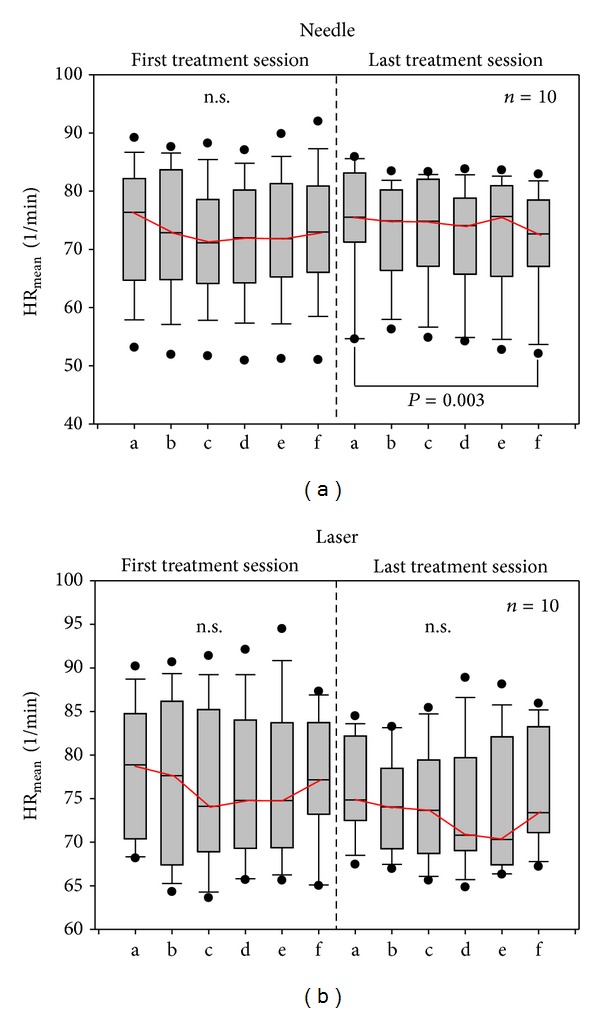
Box plots displaying the changes in mean heart rate (HR) of the 10 patients receiving needle acupuncture (a) and the 10 patients receiving laser acupuncture (b). HR had decreased significantly only after the last needle acupuncture session. The ends of the boxes define the 25th and 75th percentiles with a line at the median and error bars defining the 10th and 90th percentiles.

**Figure 5:**
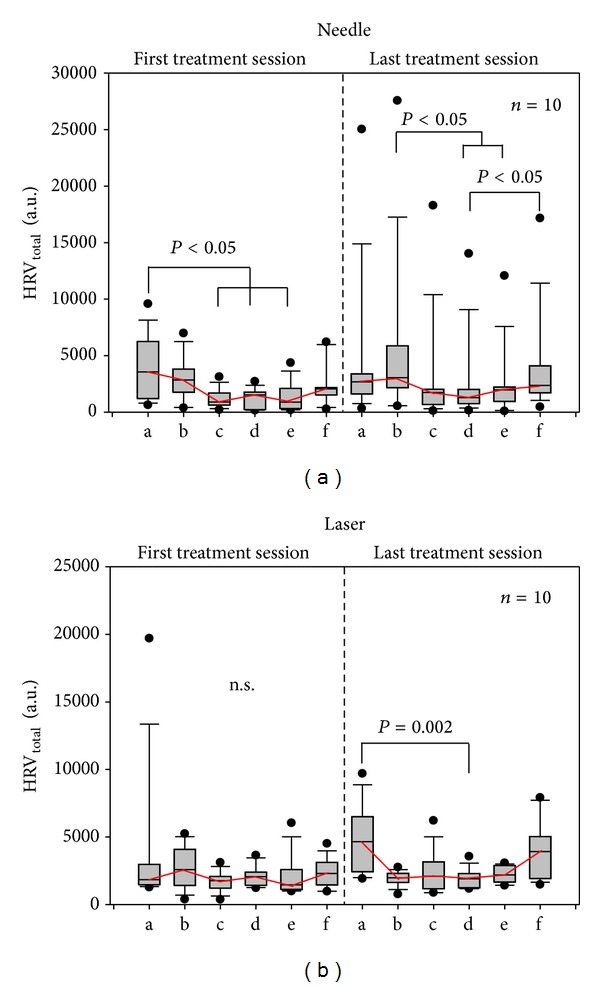
Changes in total heart rate variability (HRV). Needle and laser needle stimulation induced dynamic stimulation-related changes in total HRV in the twenty patients investigated in this study. For further explanations, see Figure 4.

Figures 4 and 5 show the mean HR and total HRV from the ECG recordings of altogether 20 patients with burnout syndrome during the six measurement phases (a–f). There was a slight decrease in HR but no significant change before, during, or after the first stimulation session with needle acupuncture. However, in the last needle acupuncture treatment session HR decreased significantly (*P* = 0.003).

In contrast to HR, HRV showed significant alterations in the first and last needle acupuncture sessions as well as in the last laser acupuncture session.

**Figure 6:**
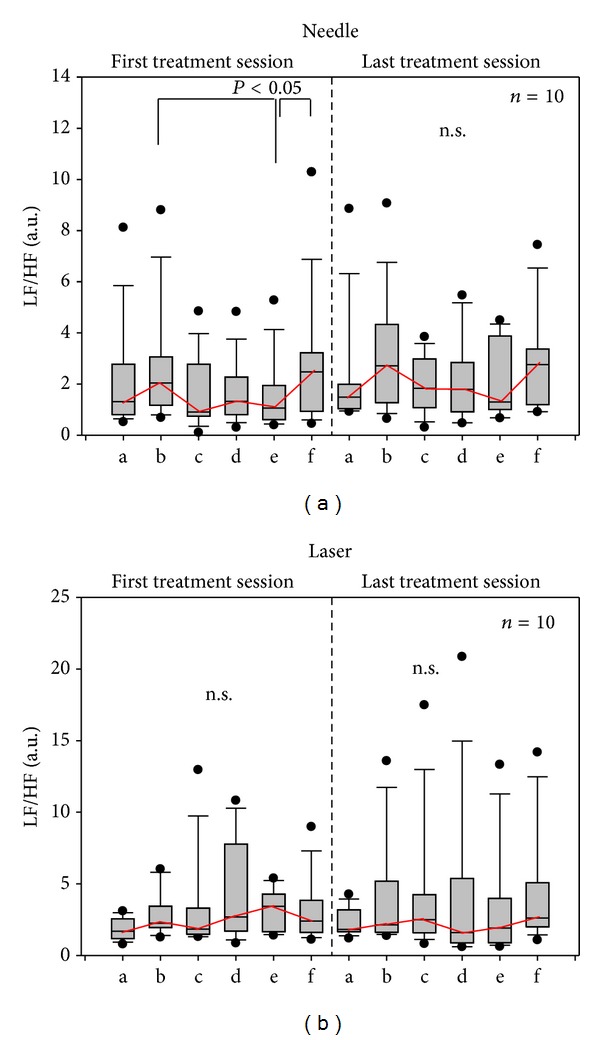
The low frequency (LF)/high frequency (HF) ratio did not change significantly during the three days of the investigation. For further explanations, see Figure 4.

Continuous HRV monitoring showed mainly no significant alterations in the LF/HF ratio during or after acupuncture stimulation (see Figure 6).

The analysis of the new HR-HRV DATS score revealed interesting results. The HR-DATS score was 0.6 during needle acupuncture treatment and 1.7 during laser needle treatment. The HRV-DATS score showed a value of 0.8 during needle treatment and 4.0 during laser needle treatment.

## 4. Discussion

Burnout can cause serious health implications. In a systematic review of the scientific literature published in 2013, burnout was found to be a risk factor for myocardial infarction, coronary heart diseases, and many other diseases [20]. Chronic stress-related disorders often fall outside the category of a “true” disease and are often treated as depression or not treated at all [20]. Symptoms of stress can be temporarily relieved with drugs, although they do not remove the cause of stress. Developing new treatment approaches therefore has priority, and invasive (needle) or noninvasive (laser needle) acupuncture is one possibility.

In 2013 a randomized, sham-controlled trial investigating acupuncture for chronic fatigue syndrome was published [21]. In this study the authors from Hong Kong saw improvements in clinical scores also after sham acupuncture. They concluded that these effects may be due to the pressure of the sham needles on the acupuncture points, in addition to the placebo effect [21]. Our present study has a completely different study design. There was no control group; we compared two different acupuncture methods in two patient groups, both with the diagnosis burnout. One method was invasive manual needle acupuncture and the other noninvasive laser needle acupuncture. Neurovegetative parameters like HR and HRV were chosen as evaluation parameters. Developing a new parameter, the so-called “dynamic acupuncture treatment score” (DATS), we were able to quantitatively describe treatment effects on the different neurovegetative responses immediately and 5 minutes after the acupuncture sessions. DATS is a simple ratio which reflects the dynamic alterations within different treatment sessions. It shows how the body (neurovegetative system) reacts to a stimulation (in our case manual needle and laser needle acupuncture). The values from the first and last sessions were compared so that one can compare changes and thus effects of the acupuncture treatment sessions. It was interesting that our new score shows stronger effects of laser needle acupuncture than of manual needle acupuncture after six treatment sessions. However, both groups had a very small sample size (10 patients each), and therefore more investigations are absolutely necessary.

In conclusion it can be stated based on new neurovegetative acupuncture treatment evaluation scores that noninvasive laser needle acupuncture, like manual needle acupuncture, has the potential to be a powerful approach for evidence-based complementary treatment of patients with burnout syndrome. Further transcontinental studies to verify or refute the preliminary findings are in progress.

## Figures and Tables

**Table 1:** Scientific literature (as of Sep 15, 2013) concerning fatigue, burnout, and chronic fatigue syndrome and complementary medical treatment methods (acupuncture and laser acupuncture).

PubMed (http://www.pubmed.gov)	Acupuncture	Laser Acupuncture
Fatigue	232	4
Burnout	3	0
Chronic Fatigue Syndrome	50	1
